# Nanomechanical hydrodynamic force sensing using suspended microfluidic channels

**DOI:** 10.1038/s41378-023-00531-1

**Published:** 2023-05-08

**Authors:** Alberto Martín-Pérez, Daniel Ramos

**Affiliations:** Optomechanics Lab, Instituto de Ciencia de Materiales de Madrid (ICMM-CSIC), 3 Sor Juana Inés de la Cruz (Madrid), E-28049 Madrid, Spain

**Keywords:** Nanofluidics, Sensors

## Abstract

Microfluidics has demonstrated high versatility in the analysis of in-flow particles and can even achieve mechanical properties measurements of biological cells by applying hydrodynamic forces. However, there is currently no available technique that enables the direct measurement and tracking of these hydrodynamic forces acting on a flowing particle. In this work, we introduce a novel method for the direct measurement of the hydrodynamic force actuating on an in-flow particle based on the analysis of the induced resonance changes of suspended microchannel resonators (SMRs). This hydrodynamic force sensitivity depends on the device used; therefore, we considered the geometry and materials to advance this dependency on the SMR resonance frequency.

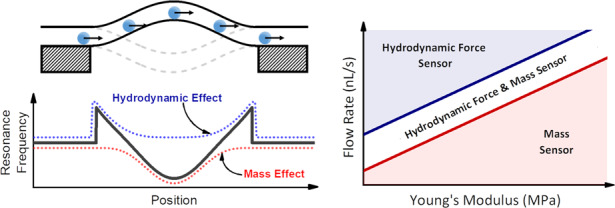

## Introduction

Numerous advances in micro- and nanotechnology necessitate the simultaneous development of tools and techniques for studying the components of the microscopic world and their interactions. In this sense, microfluidics was introduced as a revolutionary technique^1,2^, indicating the possibility of numerous experiments performed rapidly and repeatedly while consuming minimal reagent^3–5^. Accordingly, mechanical^6^ and optical^7^ microparticle analysis has been greatly benefited by microfluidics, which enables the analysis and classification of particles with extremely high throughput (up to 10^7^ particles per minute^8^) in physiological environments and at a low cost^9^. Moreover, the use of microfluidic channels presents an additional advantage of applying controlled hydrodynamic forces on a particle to characterize mechanical properties (i.e., Young’s modulus or Poisson ratio) of the in-flow particles^10^. The relevance of measuring these mechanical properties is due to their strong relationship to the chemical composition of the particles, enabling real-time particle discerning. Polymers represent a good example of the significance of studying these mechanical parameters since their stiffness depends directly on the conditions under which crosslinking occurs^11^. However, the field in which mechanical characterization shows its potential is microbiology^12,13^ since the mechanical properties of cells have been demonstrated to be intimately related to different biological processes and the development of certain diseases, such as cancer^14,15^.

**Fig. 1:**
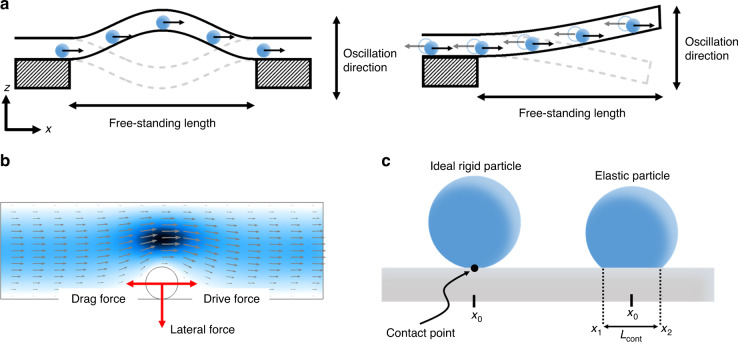
Suspended microchannel resonator devices and hydrodynamic forces. **a** Schematic of double-clamped (string-like, left) and single-clamped (cantilever-type, right) suspended microchannel resonators. Please note that the cantilever-type devices contain a U-shaped embedded channel, so the inlet and outlet of the particles are clamped. **b** Fluid velocity field inside a microchannel with a particle inside and schematic of the hydrodynamic forces acting on the particle. **c** Schematic of the contact of the particle with the microchannel wall for an ideal rigid particle (left) and an elastic particle (right)

To measure the mechanical properties of a microparticle, we have considered its response to an exerted force. However, in conventional microfluidic devices, the hydrodynamic forces applied on a particle cannot be directly measured; they can only be inferred by considering parameters such as the particle position, the velocity, and the applied flow rate. Therefore, having a technique enabling the measurement of these hydrodynamic forces actuating on the particles can potentially involve a novel step forward in the advancement of microfluidics, facilitating more accurate studies of high relevance in biomedicine or material science. In this sense, the suspended microchannel resonator (SMR) approach is the most promising candidate since it merges the advantages of nanomechanical resonators and microfluidics^16–19^. Nanomechanical resonators are currently considered one of the most promising sensor devices, showing unprecedented mass sensitivity of a single atom^20^ and force sensitivity of a single spin^21^. Their canonical image consists of a free-standing structure that can mechanically oscillate (usually in a flexural mode). The resonance frequency of these systems depends on different physical parameters^22–24^ (e.g., mass, geometry, force, and spin) and chemical parameters^24–26^ (e.g., humidity and presence of chemical species); thus, by tracking its variations, the changes in these physical/chemical parameters can be measured with extremely high precision and accuracy. SMR devices consist of a suspended structure (mechanical resonator) clamped by one (cantilever-type) or two ends (string-type) inside, where a microfluidic channel is integrated (Fig. 1a). Consequently, these structures can oscillate in flexural modes, enabling nanomechanical sensing, and the integrated microchannel enables the controlled delivery of particles into the suspended area. Notably, for SMR devices with single-clamped geometries, the integrated microchannel has a “U” shape to allow the liquid to flow through the suspended area. Therefore, when a particle passes through the suspended region, it produces a change in the mechanical properties of the resonator, and consequently, the resonance frequency changes. Tracking these frequency shifts in the SMR devices has been demonstrated to be useful for measuring the physical properties of particles such as mass^16^, density^27^, or volume^28^.

Moreover, previous studies on SMR devices have demonstrated that they are not only excellent mass sensors^16,17,28,29^ for in-flow particles but can also measure particle optical absorption^30^, hydrodynamic properties^31^, and hydrostatic forces^32,33^; however, no previous studies on this topic have measured the hydrodynamic force actuating on a flowing particle. In our study, we discuss the possibility of measuring this force using these devices and the conditions needed to obtain this measurement.

## Results

### Suspended microchannel resonators for mass and hydrodynamic force sensing

When a particle is introduced into the stream of a microfluidic channel, a hydrodynamic force acts on it, causing its displacement. This hydrodynamic force can be discomposed into the sum of two different force components: a parallel and an orthogonal force to the flow (Fig. 1b). The parallel force is eventually balanced when the drag and drive forces have the same magnitude (Fig. 1b), resulting in the particle moving with a constant velocity in the flow direction^31^. Moreover, the orthogonal force (also known as the lift force) produces a lateral displacement of the particle in the orthogonal direction, such that the particle usually occupies an equilibrium position where this orthogonal force is balanced^34^. Previous studies have demonstrated that under certain conditions, which include SMR devices, this equilibrium position cannot be reached inside the channel, and the particle eventually precipitates on the channel wall while moving with a constant velocity in the parallel direction^30,34,35^. Therefore, hereinafter, we will assume that the particle is always precipitated on the resonator’s wall.

When the particle is in direct contact with the channel wall, the hydrodynamic orthogonal force has a net value that can be calculated by the asymmetries of the integral of the fluid’s stress tensor (*σ*_*ij*_)^36^ over the surface of the particle ${F}_{z}\,=\oint- {\sigma}_{zi}{dS}$. For this purpose, the first order is assumed where the particle’s diameter is much smaller than the inner channel diameter; therefore, the parabolic flow profile inside the inner channel remains unperturbed by the presence of the particle, while its shape can approach a cube (Eq. 1, see materials and methods for further details on this calculation). Despite the simplicity of this approach, recent works have demonstrated that experimental conditions reproduce the simulation results for particles whose diameters are smaller than ¾ of the channel diameter^31^,

1 $$F_z = \frac{16\mu r_{{{part}}^{3}}}{{\pi r_{{in}}{\,}^{4}}}q$$

where *μ* is the viscosity, *r*_part_ is the particle radius, *r*_in_ is the inner channel radius, and *q* is the flow rate.

In regular channels, this orthogonal force determines the position of the particle; however, in the suspended region of SMR devices, this applied force will locally change the stiffness of the resonator, resulting in an increase in the resonance frequency^37^. Therefore, these SMR devices can be used to perform a direct measurement of this hydrodynamic force. To theoretically study the effect of this hydrodynamic force on the resonance frequency of the SMR device, we utilize the Galerkin discretization of the resonator into their harmonic components, using an effective value of mass (*m*_eff_) and spring constant (*k*_eff_)^38^. As the particle passes through the suspended area, it will change either the effective mass or the effective spring constant of the resonator. The variation in the effective mass of the resonator produced by the particle (mass effect) has been widely studied in previous works on SMR devices^16,17,30^, demonstrating that the particle can be considered a punctual mass changing the resonator effective mass depending on its position on the resonator, as shown in Eq. 2 (see materials and methods for further details):

2 $$m_{{\rm{eff}},n} = m_0 + m_b\psi _n^2\left( {x_0} \right)$$

where *m*_0_ is the mass of the resonator, *m*_*b*_ is the buoyant mass of the particle, *x*_0_ is the position of the particle, and *ψ*_*n*_ is the shape of the n-th oscillation mode obtained by the Euler–Bernoulli beam theory^39^ (the model conventionally used in SMR devices).

On the other hand, to calculate the change produced in the effective spring constant caused by the hydrodynamic force applied by the particle on the resonator’s wall (hydrodynamic force effect), we consider this hydrodynamic force as an applied pressure on the resonator’s wall ($F_z/A_{\rm{cont}}$, being *A*_cont_ particle’s contact area, Fig. 1c). Therefore, the effective spring constant can be calculated as follows

3 $$k_{eff} = IE\frac{{\alpha _n^4}}{{L^3}} + I\;L_{cont}\frac{{F_z}}{{A_{cont}}}\psi _n^{\prime\prime 2}\left( {x_0} \right)$$

where *I* is the second moment of inertia, *E* is Young’s modulus of the resonator wall, *α*_*n*_ is the eigenvalue of the oscillation mode, *L* is the length of the suspended area, and *L*_cont_ is the contact length.

Once the effective mass and stiffness are calculated, the resonance frequency of the device can be obtained by using the harmonic oscillator approximation^38^.

4 $$f_n = \frac{1}{{2\pi }}\sqrt {\frac{{I\left( {E{\textstyle{{\alpha _n^4} \over {L^3}}} + {\textstyle{F \over {A_{\rm{cont}}}}}L_{\rm{Cont}}\psi _n^{\prime \prime 2}\left( {x_0} \right)} \right)}}{{m_0 + m_b\psi _n^2\left( {x_0} \right)}}}$$

Considering that the frequency shift caused by the passing particle (∆*f*_*n*_) is much smaller than the initial resonance frequency (*f*_*n*,0_), we can make a linear approximation as follows:

5 $$\frac{{{\Delta}f_n}}{{f_{n,0}}} \approx - \frac{1}{{2m_0}}m_b\psi _n^2\left( {x_0} \right) + \frac{{L_{\rm{Cont}}L^3}}{{\alpha _n^4A_{\rm{cont}}E}}F_z\,\psi _n^{\prime\prime 2}\left( {x_0} \right)$$

Notably, these expressions can be used either for single (cantilever) or doubly clamped (string) resonators at any *n* flexural mode simply by replacing the corresponding eigenvalue, *α*_*n*_.

### Decoupling mass and force effects

**Fig. 2:**
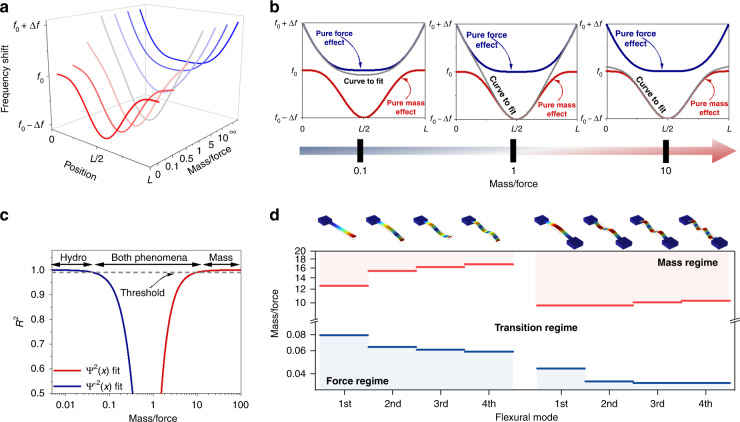
Mass and force effects. **a** Frequency shift calculated for different mass/force ratios. **b** Frequency shift curves for different mass/force ratios (gray lines) compared to a pure force model (blue) and a pure mass model (red). **c** Coefficient of determination calculated as a function of the mass/force ratio. **d** Limit of the mass and force regimes calculated for the first four flexural modes in a cantilever and a string SMR device

When a particle passes through the suspended region, it produces a shift in the resonance frequency, which depends on its position, its mass, and the hydrodynamic force (Eq. 5 and Fig. 2a). Therefore, in a real experiment, the mass and hydrodynamic force could be obtained by fitting the frequency shift to Eq. 5. From Eq. 5, it follows that this resonance frequency shift is a linear combination of two orthogonal functions: $\psi ^2\left( x \right)$ and $\psi ^{\prime\prime 2}\left( x \right)$. Each of these two functions is related to one of the two phenomena that govern the change in the resonance frequency, with the function $\propto \psi ^2\left( x \right)$ related to the mass variation (mass effect) and the function $\propto \psi ^{\prime\prime 2}\left( x \right)$ related to the applied hydrodynamic force. Depending on the experimental conditions (density and radius of the particle as well as flow rate), one of these two effects can be much larger than the other one (Fig. 2b), which results in the detection of only one of them in a real experiment. This is the case for previous studies on SMR devices^16,17,30,40^, in which only the mass effect has been measured, implying that the force effect is negligible for the experimental conditions typically used. To determine which conditions are optimal to measure the hydrodynamic force effect, we study both limits of Eq. 5 as a function of the mass/force ratio (see materials and methods for further details): $\psi ^2\left( x \right)$, pure mass effect, Fig. 2b; and $\psi ^{\prime\prime 2}\left( x \right)$, pure force effect, Fig. 2b. For this purpose, we perform two nonlinear fittings for different mass/force ratios, and we calculate the coefficient of determination (*R*^2^, Fig. 2c) for each fitting. This coefficient enables the determination of the effect (mass or force effect) that can be neglected, with *R*^2^ = 1 being the ideal case with no error. As expected, for the pure mass effect, the coefficient of determination approaches 1 when the mass/force ratio approaches infinity; for the pure force effect, the *R*^2^ approaches 1 when the mass/force approaches 0. However, in real experiments, due to the noise level, obtaining an *R*^2^ = 1 when fitting the collected data to a function is improbable. For this reason, hereinafter, we set the criterion such that we can neglect one of the two effects (mass or force) without making a significant error as long as *R*^2^ > 0.99 for one of the two functions. This criterion allows us to set 3 different regimes depending on the mass/force ratio: mass regime (i.e., the force effect is neglected), force regime (i.e., the mass effect is neglected), and transition regime (i.e., both effects are considered). Therefore, the limit for both the mass and transition regimes is found to be a mass/force ratio of ~10, while the limit between the force and transition regimes is approximate ~0.1 (Fig. 2c). Notably, the development of these three regimes provides a way to measure either the particle mass (as done in previous works) or the hydrodynamic force by switching the experimental conditions. Moreover, within the transition regime, the mass and hydrodynamic force can be measured simultaneously if the frequency of two oscillation modes is tracked^41,42^. Figure 2d shows the limits between the mass and force regimes for single- and double-clamped SMR devices. From this plot, it can be deduced that reaching the transition and the force regime for the single-clamp SMR device requires a slightly higher mass/force ratio than that in the case of the double-clamped resonator; thus the cantilevered SMR device can be potentially more efficient for hydrodynamic force sensing purposes. Moreover, Fig. 2d shows that for higher oscillation modes, the mass limit increases while the force limit decreases; therefore, it is slightly more difficult to reach either the pure mass or force regimes.

### Conditions for force sensing

Due to the established limits under which the effect of the hydrodynamic force cannot be neglected, we study which specific conditions should be used in real experiments for its measurement. For this purpose, we obtain an analytical expression for the mass/force ratio by combining Eq. 5 and Eq. 1 (see appendix for further details). Since the value of the mass/force ratio depends on the specifications of the SMR device, the analyzed sample, and the experimental conditions (flow rate and oscillation mode) used, we can rearrange the analytical expression of the mass/force ratio to obtain it as the product of these three factors as shown below:

6 $$\frac{\rm{mass}}{\rm{force}} = \frac{{\pi ^2}}{6}\varepsilon _{\rm{dev}}\varepsilon _{\rm{sam}}\varepsilon _{\rm{opm}}$$

7 $$\varepsilon _{opm} = \frac{{\alpha ^4}}{q}\left( {\frac{{\psi \left[ {x_{\max ,1}} \right]}}{{\psi \prime\prime \left[ {x_{\max ,2}} \right]}}} \right)^2$$

8 $$\varepsilon _{\rm{sam}} = \frac{{\left| {{\Delta}\rho } \right|A_{\rm{cont}}}}{{\mu L_{\rm{Cont}}}}$$

9 $$\varepsilon _{\rm{dev}} = \frac{{r_{\rm{in}}^4E}}{{m_0L^3}}$$

where ∆*ρ* is the mass density contrast (the mass density difference between the particle and the carrier liquid), *ε*_dev_ is the device factor, *ε*_sam_ is the sample factor and *ε*_opm_ is the operation mode factor.

**Fig. 3:**
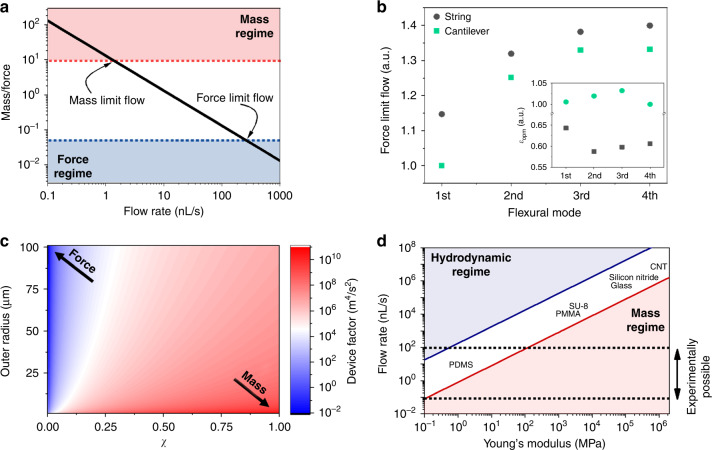
Optimizing the devices. **a** Mass/force ratio as a function of the flow rate calculated for a string-type SMR. **b** Force limit flow for different geometries and oscillation modes. Inset. Operation mode factor for different oscillation modes and geometries. **c** Device factor as a function of the geometry. For this calculation, the ratio between the outer diameter and the resonator length was kept constant at 1/10. **d** Regime diagram as a function of flow rate and Young’s modulus. The solid lines show the mass and force flow limits

Since the hydrodynamic force is directly proportional to the flow rate, the operation mode factor (*ε*_opm_) enables direct tuning of the mass/force ratio. Therefore, there is a certain flow rate at which the force (or mass) regime is reached (Fig. 3a); hereinafter, it is called the limit flow. Since this parameter depends on the oscillation mode (inset in Fig. 3b), the limit flow will change for different modes and geometries, as shown in Fig. 3b. Notably, although the operation mode factor is lower for the double clamped geometry than for the single clamped geometry, the force limit flow is lower for the single clamped geometry due to a higher limit mass/force ratio. Moreover, the limit flow is increased with the mode number, *α*, while the difference between the flow limit in both geometries is reduced.

On the other hand, the sample factor depends either on the properties of the flowing particles or the carrier liquid. From this factor, it can be concluded that the mass/force ratio can be minimized by using rigid particles, reducing the contact area and consequently $A_{\rm{cont}}/L_{\rm{Cont}} \to 0$. Moreover, this sample factor facilitates a pure force regime by switching the density contrast to zero, where the buoyant mass and, therefore, the mass effect will be null. The density contrast can be experimentally tuned by controlling the concentration of solvents in the carrier liquid (usually water); however, this is not always possible, especially in biological samples, where changing the salt concentration in the carrier liquid can result in an undesired change in the physical properties of the analytes^10^.

Alternatively, the device factor facilitates the optimization of the resonator design to reach the force regime. This factor depends on the geometry and on the mechanical properties of the structural materials (mass density and Young’s modulus); thus, choosing the optimal material and geometry will facilitate reaching the force regime. By choosing a cylindrical hollow resonator, the device factor (*ε*_dev_) can be expressed as follows:

10 $$\varepsilon _{\rm{dev}} = \frac{{\chi ^4r_{\rm{ext}}^2E}}{{\pi \left( {\rho _w\left[ {1 - \chi ^2} \right] + \rho _l\,\chi ^2} \right)}}$$

where *r*_ext_ is the external radius of the SMR device, *χ* is the wall ratio, i.e., the ratio between the inner and outer diameters ($r_{in}/r_{ext}$), *ρ*_*w*_ the mass density of the wall, and *ρ*_*l*_ is the mass density of the carrier liquid.

When plotting the device factor as a function of the external radius and the wall ratio (Fig. 3c.) and to maximize the force effect, these devices need to be fabricated using larger outer diameters and smaller inner diameters. However, if the resonator has a small outer diameter with a thin wall, the mass effect will be maximized (Fig. 3c).

The most important/critical parameter in the device factor is Young’s modulus. A softer material correlates to a higher hydrodynamic force sensitivity of the device. Figure 3d shows the different operating regimes as a function of the flow rate and Young’s modulus. The mass and the force limit flows are represented as red and blue solid lines. Within a range of the typical flow rates used in this application (<100 nL/s), it is necessary to use materials with a very low Young’s modulus (~1 MPa) to reach the force regime for the typical device dimensions (suspended length of 500 μm, 50 μm outer diameter, 40 μm inner diameter and particles with diameters ~5 μm). Among all the common materials used in MEMS/NEMS and microfluidics, poly dimethyl siloxane (PDMS) is the most promising candidate for use as a hydrodynamic force sensor due to its Young’s modulus. Additionally, we deduce that the force effect has not been previously measured because the materials used in the fabrication of these devices (silicon^16^, silicon oxide^43^, silicon nitride^32^, or fused silica^17,38,39,44^) need a flow rate at least three orders of magnitude higher than the usual experimental values.

## Discussion

In this work, we have proposed using suspended microchannel resonators as sensors for the hydrodynamic force acting on flowing particles. We analytically studied the resonance frequency shift of SMRs caused by a passing particle, showing that it could be understood as the linear combination of two different phenomena: (1) the added mass caused by the particle’s buoyant mass (mass effect) and (2) the change in the stiffness caused by the hydrodynamic force transmitted (force effect) to the microchannel walls. Moreover, we demonstrated that different experimental conditions could be varied to minimize one effect or another; the most significant parameter was the flow rate. In this sense, we pinpointed the maximum/minimum flow rate needed to neglect the force or the mass effect. Eventually, we discussed how suspended microchannel resonators should be fabricated for future experiments to measure their hydrodynamic force; it was determined that these devices needed to have thick walls when fabricated in a soft material (e.g., PDMS).

## Materials and methods

### Calculation of the hydrodynamic orthogonal force

Considering a cylindrical pipe and a laminar flow regime, the flow velocity (*U*) will follow a parabolic profile (Eq. A.1). Then, by applying the Poiseuille law, we can substitute the value of *u*_0_ (fluid velocity at the axis of the pipe) by an expression depending on the flow rate (Eq. A.2).

A.1 $$\vec U = u_0\left( {1 - \frac{{y^2 + z^2}}{{r_{in}^2}}} \right)\overrightarrow {u_x}$$

A.2 $$u_0 = \frac{{2q}}{{\pi r_{in}^2}}$$

A.3 $$\sigma _{ij} = \mathop {\sum}\limits_{i,j} {\left[ {p\delta _{ij} + \mu \left( {\frac{{dU_i}}{{dx_j}} + \frac{{dU_j}}{{dx_i}}} \right)} \right] = \frac{{2u_0}}{{r_{in}^2}}\left( {\begin{array}{*{20}{c}} 0 & y & z \\ y & 0 & 0 \\ z & 0 & 0 \end{array}} \right)}$$

where *p* is the pressure and *δ*_*ij*_ is the Krönecker delta.

Eventually, the stress tensor is calculated (Eq. A.3). This stress tensor is symmetric for all opposite faces of the cube with the exception of the top and bottom faces, producing the orthogonal force, as shown in Eq. 1. Please note that since the particle diameter is much smaller than the pipe length, we can consider the pressure difference to be null between the different faces of the particle; thus, we can neglect this term.

### Calculation of the frequency shift

The frequency shift caused by a passing particle is calculated considering the resonance frequency of the SMR device as the resonance frequency of a harmonic oscillator (Eq. A.4)^38^. The effective mass when a particle crosses the suspended region is calculated considering the particle as a punctual mass at a certain position (*x*_0_, Eq. A.5). For the calculation of the effective spring constant (Eq. A6), the punctual particle approach (Fig. 1c) is not considered because in that case, the equations diverge. Therefore, ae simpler way to consider how the hydrodynamic force changes along the x-axis is assuming it is 0 outside of the contact area while it remains constant within the contact area.

A.4 $$f_n = \frac{1}{{2\pi }}\sqrt {\frac{{k_{eff,n}}}{{m_{eff,n}}}}$$

A.5 $$m_{eff,n} = \mathop {\int}\limits_0^L {\left( {\rho A + m_b\delta \left[ {x - x_0} \right]} \right)\psi _n^2\left( x \right)dx = m_0 + m_b\psi _n^2\left( {x_0} \right)}$$

A.6 $$\begin{array}{l}k_{{{eff}},n} = \mathop {\int}\nolimits_0^L {I\left( {E + \frac{{F_z\left( x \right)}}{{A_{{cont}}}}} \right)\psi _n{\,}^{{\prime\prime} 2}\left( x \right)dx = \mathop {\int}\nolimits_0^L {IE\psi _n{\,}^{{\prime\prime} 2}\left( x \right)dx + \mathop {\int}\nolimits_{x_1}^{x_2} {I\frac{{F_z}}{{A_{{cont}}}}\psi _n{\,}^{{\prime\prime} 2}\left( {x_0} \right)dx} } } \\ \qquad\,\,\approx \,IE\frac{{\alpha _n{\,}^4}}{{L^3}} + I\;L_{{cont}}\frac{F}{{A_{{cont}}}}\psi _n{\,}^{{\prime\prime} 2}\left( {x_0} \right)\end{array}$$

Please note that to solve the integral in Eq. A.6, we have considered the following: given *L* ≫ *L*_cont_, then $\psi _n^{\prime\prime 2}\left( x \right) \approx \psi _n^{\prime\prime 2}\left( {x_0} \right)$ within the contact length.

### Calculation of the mass/force ratio

To calculate the analytical expression of the mass/force ratio (Eq. A.7), we calculate the quotient between the absolute value of the mass term (${\textstyle{{m_b} \over {2m_0}}}\psi _n^2\left( x \right)$) and the force term (${\textstyle{{L_{\rm{Cont}}L^3} \over {\alpha _n^4E}}}F_z\psi _n^{{\prime\prime} 2}\left( x \right)$), as shown below:

A.7 $$\frac{{mass}}{{force}} = \frac{{\alpha _n{\,}^4\,A_{{cont}}E}}{{2m_0L_{{Cont}}L^3}}\frac{{m_b}}{F}\left( {\frac{{\psi _n\left( {x_0} \right)}}{{\psi _{{n}^{\prime\prime}} \left( {x_0} \right)}}} \right)^2$$

Then, considering that the buoyant mass for a spherical particle is defined as $m_b = {\textstyle{4 \over 3}}\pi {\Delta}\rho r_{\rm{part}}^3$ and the expression of the orthogonal hydrodynamic load (Eq. 1), we can convert the mass/force ratio into the following expression:

A.8 $$\frac{{mass}}{{force}} = \frac{{\pi ^2}}{6}\left( {\frac{{r_{{in}}{\,}^4E}}{{m_0L^3}}} \right)\left( {\frac{{\left| {{\Delta}\rho } \right|A_{{cont}}}}{{\mu L_{{Cont}}}}} \right)\left( {\frac{{\alpha ^4}}{q}\left[ {\frac{{\psi \left[ {x_{max ,1}} \right]}}{{\psi ^{\prime\prime} \left[ {x_{max ,2}} \right]}}} \right]^2} \right)$$

Please note that the expression has been rearranged such that each parenthesis contains variables that depend on only one aspect of the experiment. Therefore, the first parenthesis contains variables that depend on the SMR device’s structure (*ε*_dev_, Eq. 9), the second parenthesis contains variables that depend on the sample to be analyzed (*ε*_sam_, Eq. 8), and the third parenthesis contains values that depend on the way the SMR device is operated (*ε*_*opm*_, Eq. 7).
